# Hybrid Support Vector Regression and Autoregressive Integrated Moving Average Models Improved by Particle Swarm Optimization for Property Crime Rates Forecasting with Economic Indicators

**DOI:** 10.1155/2013/951475

**Published:** 2013-05-23

**Authors:** Razana Alwee, Siti Mariyam Hj Shamsuddin, Roselina Sallehuddin

**Affiliations:** Soft Computing Research Group, Faculty of Computing, Universiti Teknologi Malaysia, 81310 Skudai, Johor, Malaysia

## Abstract

Crimes forecasting is an important area in the field of criminology. Linear models, such as regression and econometric models, are commonly applied in crime forecasting. However, in real crimes data, it is common that the data consists of both linear and nonlinear components. A single model may not be sufficient to identify all the characteristics of the data. The purpose of this study is to introduce a hybrid model that combines support vector regression (SVR) and autoregressive integrated moving average (ARIMA) to be applied in crime rates forecasting. SVR is very robust with small training data and high-dimensional problem. Meanwhile, ARIMA has the ability to model several types of time series. However, the accuracy of the SVR model depends on values of its parameters, while ARIMA is not robust to be applied to small data sets. Therefore, to overcome this problem, particle swarm optimization is used to estimate the parameters of the SVR and ARIMA models. The proposed hybrid model is used to forecast the property crime rates of the United State based on economic indicators. The experimental results show that the proposed hybrid model is able to produce more accurate forecasting results as compared to the individual models.

## 1. Introduction

Quantitative forecasting methods are classified into causal and time series models. The causal models are based on the relationship between the variable to be forecasted and independent variables. A linear relationship is typically used in the causal models. Regression, econometric models, and input-output models are examples of some of the causal models. The time series models are models that use historical data to estimate the future which can be categorized into linear and nonlinear models. Most of the linear models are statistical models such as exponential smoothing, moving average, and autoregressive integrated moving average (ARIMA). However, the nonlinear models consist of statistical models such as bilinear models, the threshold autoregressive (TAR) models, and autoregressive conditional heteroscedastic (ARCH) as well as nonstatistical models such as artificial neural networks (ANN) and support vector regression (SVR). 

The causal models such as regression and econometric models are commonly used in crime rates forecasting. The causal models can describe the causal relationship between the crime variable and other explanatory variables. However, the development of the causal models is quite complex and requires theoretical assumptions about the relationship between the explanatory variables. Therefore, the time series model has been considered as a promising alternative tool for crime rates forecasting. The application of time series models for crime rates forecasting is still scarce. Standard time series models usually require a substantial number of observations. However, insufficient crime data makes the standard time series models less suitable for crime rates forecasting. Therefore, a new model that suits small data set is needed to improve the crime rates forecasting performance.

There are two types of time series models, namely, linear and nonlinear models. The linear models can only model the linear relationship, while the nonlinear models only model the nonlinear relationship. In the literature, there is no single model that can predict well in all conditions. Therefore, many researchers have used a hybridization of linear model with nonlinear model as an approach to time series forecasting [1]. The hybrid linear and nonlinear models are not only capable of modeling the linear and nonlinear relationships, but are also more robust to changes in time series patterns [2]. Artificial neural networks (ANNs) and support vector regression (SVR) are two nonlinear models usually being employed, while ARIMA, seasonal autoregressive integrated moving average (SARIMA), autoregression (AR), exponential smoothing, moving average, and multiple linear regression are usually used to represent linear model in hybridization of linear and nonlinear model. Several examples of hybrid time series models that have been proposed in the literature are ARIMA and ANN [1–16], ARIMA and SVR [17–23], seasonal autoregressive integrated moving average (SARIMA) and SVR [24, 25], autoregression (AR) and ANN [26], exponential smoothing and ANN [27], ARIMA and genetic programming (GP) [28], exponential smoothing, ARIMA and ANN [29], and multiple linear regression (MLR) and ANN [30]. 

A hybridization of ARIMA and ANN models as linear and nonlinear model is extensively studied by researchers since it produces promising results. However, this hybridization requires sufficient data to produce a good model. Furthermore, ANN models suffer from several problems such as the need for controlling numerous parameters, uncertainty in solution (network weights), and the danger of over fitting. Support vector regression (SVR) was proposed by Vapnik [31] in order to overcome the drawback of ANN. SVR is a nonlinear model to solve regression problems and has been used by researchers as an alternative model to ANN [17–23]. A hybridization of ARIMA and SVR has been successfully applied in time series forecasting such as stock market [17, 20], electricity price [22], and power load [18, 19]. There are four factors that contributed to the success of SVR which are good generalization, global optimal solution, the ability to handle nonlinear problems, and the sparseness of the solution. This has made SVR a robust model to work with small training data, nonlinear, and high-dimensional problems [32]. Despite the advantages, SVR also has some limitations. For example, SVR model parameters must be set correctly as it can affect the regression accuracy. Inappropriate parameters may lead to overfitting or underfitting [33]. Genetic algorithm (GA) and particle swarm optimization (PSO) are among the approaches that have been used by researchers to estimate the SVR parameters. However, PSO is easier to implement as compared to GA, because it does not require evolution operators such as crossover and mutation [34]. As for linear model, ARIMA is the preferred choice by researchers for hybridization with nonlinear model due to its ability to model various smoothing models such as simple autoregressive (AR), a simple moving average (MA), and a combination of AR and MA (ARMA model) [23]. ARIMA has high forecasting performance when the data set is large and linear. However, it is not robust for small data sets and nonlinear data. Therefore, several improvements have been proposed to improve the performance of ARIMA [35]. Asadi et al. [36] suggested the use of PSO to estimate the parameters of ARIMA model for small data sets. 

Most hybrid models use a sequence of linear and nonlinear models, but there are also hybrid models that use a sequence of nonlinear and linear models. It depends on which component is more dominant, either linear or nonlinear. The dominant component needs to be modeled first. However, the linear and nonlinear components in the data can have interaction. It is quite difficult to determine which component is more dominant. Modeling linear patterns using linear model will change the nonlinear patterns, and vice versa. However, in comparison with ANN model, the SVR is able to keep the linear components undamaged [22]. A hybridization of SVR and ARIMA model with a sequence of nonlinear and linear models has been found to outperform the existing neural network approaches, traditional ARIMA models, and other hybrid models, such as ARIMA and ANN (with a sequence of linear and nonlinear models and a sequence of nonlinear and linear models), as well as ARIMA and SVR (with a sequence of linear and nonlinear models) [22]. Therefore, this study uses a sequence of nonlinear and linear models by combining SVR with ARIMA model.

 The hybrid linear and nonlinear model has never been used for crime rates forecasting. Most of the models used for crime rates forecasting are linear. As for real-world data, crime rates may have linear and nonlinear components, where the use of linear model may not be adequate to forecast the crime rates. Therefore, this study attempts to propose a hybrid time series model that can work well with limited data that consist of both linear and nonlinear components. SVR is used as nonlinear model and ARIMA is employed as linear model. First, the SVR is used to model the nonlinear component. After that, the remaining from the SVR model, which represents the linear component, is modeled by using ARIMA. In order to overcome the drawbacks of the SVR and ARIMA model, particle swarm optimization (PSO) is used to estimate the parameters of SVR and ARIMA models. PSO has the ability to escape from local optima, easy to implement, and has fewer parameters to be adjusted [34]. There are several factors that influence the property crime rates, among these are economic indicators. Therefore, this study uses three economic indicators, namely, unemployment rate, gross domestic product, and consumer price index as inputs to the proposed hybrid model.

 The remainder of this study is organized as follows. Related work on crimes and economic indicators is first discussed in Section 2. In Section 3, brief explanations on the support vector regression, ARIMA, particle swarm optimization, and the proposed hybrid model are described.  Section 4 describes the data set and model evaluation employed in this study. The determination parameters of model and the analysis of the results are presented in Sections 5 and 6, respectively. Finally, a brief conclusion is drawn in Section 7. 

## 2. Related Work on Crimes and Economic Conditions

Economic conditions are often considered to be related to crimes, especially property crimes. In the literature, many studies have been done by researchers in order to relate the economic conditions with property crimes. The unemployment rate is often selected by the researchers in their studies to represent the economic conditions [37]. A study using a country level panel data set from Europe found that unemployment has a positive influence on property crimes [38]. Meanwhile, another study based on UK annual regional data has discovered that unemployment is an important explanatory variable for crimes motivated by economic gain [39]. Results produced by some other studies also found significant relationship between the unemployment and property crimes. Among the findings is that motor vehicle theft is significantly associated with the unemployment rate [40] and is also cointegrated with male youth unemployment [41]. Another finding shows that unemployment has a positive effect on burglary, car theft, and bike theft [42]. 

 Unemployment, especially among youth and young adults, is also found to influence crimes. According to a study on the United States arrest data, unemployment has a positive relationship with theft crimes among youth and young adults (16–24 years) [43]. Another study investigated the relationship between crime with male adult (26–64 years) and youth (16–25 years) unemployment in Britain [44]. The results indicate that youth unemployment and adult unemployment are both significantly and positively related to burglary, theft, fraud, and forgery as well as total crime rates. 

 In addition to unemployment, other economic indicators such as consumer price index, gross domestic product, and consumer sentiment index were also studied by the researchers to examine the relationship between economic conditions with crimes. Several researchers used the consumer price index to measure the inflation [45, 46]. Inflation reduces the purchasing power and increases the cost of living. A study on the impact of inflation rate on crime in the United States using the modified Wald causality test found that the crime rate is co integrated with inflation and unemployment rates [45]. Further, another study which examined the linkages between inflation, unemployment, and crime rates in Malaysia revealed that inflation and unemployment are positively related to the crime rate [46]. Meanwhile, for gross domestic product, a study to explain the relationship between national crime rates with social and economic variables has found that robbery and homicide have significant negative relationship with gross domestic product [47]. 

As a conclusion, the economic conditions do have an influence on the property crime rates. Therefore, this study attempts to employ the economic conditions to forecast the property crime rates. The economic conditions will be represented by three economic indicators, namely, unemployment rate, consumer price index, and gross domestic product. These economic indicators are used as input to the proposed hybrid model for forecasting property crime rates.

## 3. Methodology

In this section, explanations on support vector regression, ARIMA, and particle swarm optimization are summarized as a basis to describe the proposed hybrid model.

### 3.1. Support Vector Regression (SVR)

Support vector regression (SVR) is a nonlinear model to solve regression problems. SVR training process is similar to solving the linearly constrained quadratic programming problems that provides a unique optimal value and there is no local minimum problem. The solution is sparse, as only essential data are used to solve the regression function. Lagrangian multipliers are introduced to solve the problem. The SVR model is given by formula [48]

(1) $$\begin{array}{c} f\left(x\right)=\left(z\cdot \phi \left(x\right)\right)+b, \end{array}$$

where **z** is weight vector, *b* is a bias value, and *ϕ*(**x**) is a kernel function. SVR used *ε*-insensitivity loss function which can be expressed as formula

(2) $$\begin{array}{c} L_{\varepsilon }\left(f\left(x\right)-y\right)=\{\begin{array}{cc} \left|f\left(x\right)-y\right|-\varepsilon , & \text{if}\;\;\left|f\left(x\right)-y\right|\geq \varepsilon ,\\ 0, & \text{otherwise}, \end{array} \end{array}$$

where *ε* is the region for *ε*-insensitivity. Loss is accounted only if the predicted value falls out of the band area. The SVR model can be constructed to minimize the following quadratic programming problem:

(3) min⁡:12zTz+C∑i(ξi+ξi∗),subjected  to yi−zTxi−b≤ε+ξi,zTxi+b−yi≤ε+ξi∗,ξi,ξi∗≥0,

where *i* = 1,2,…, *n* is the number of training data, (*ξ*_*i*_ + *ξ*_i_*) is the empirical risk, (1/2)**z** ^*T*^ **z** is the structure risk preventing overlearning and lack of applied universality, and *C* is the regularization parameter. After selecting proper regularization parameter (*C*), width of band area (*ε*) and kernel function (*K*), the optimum of each parameter can be resolved through Lagrange function. The commonly used kernels are linear kernel, polynomial kernel, radial basis function (RBF), or Gaussian kernel and sigmoid kernel. Formulas (4), (5), (6), and (7) are the equation for linear kernel, polynomial kernel, RBF kernel [49], and sigmoid kernel [50], respectively. Consider

 linear kernel,
  (4) $$\begin{array}{c} K\left(x_{i},x_{j}\right)=x_{i}^{T}x_{j}, \end{array}$$
 polynomial kernel,
  (5) $$\begin{array}{c} K\left(x_{i},x_{j}\right)={\left(1+x_{i}\cdot x_{j}\right)}^{d}, \end{array}$$
 RBF kernel,
  (6) $$\begin{array}{c} K\left(x_{i},x_{j}\right)=\exp\left(-\gamma {||x_{i}-x_{j}||}^{2}\right), \end{array}$$
 sigmoid kernel,
  (7) $$\begin{array}{c} K\left(x_{i},x_{j}\right)=\tanh\left[v\left(x_{i},x_{j}\right)+\alpha \right]. \end{array}$$

The type of kernel function influences the parameters of SVR kernel. The kernel function and parameters of SVR kernel function should be set properly because it can affect the regression accuracy. Inappropriate parameters may lead to over-fitting or under-fitting [33]. This study uses the RBF kernel function because it suits most forecasting problems [51]. The RBF kernel is also effective and has fast training process [52]. For the RBF kernel function, there are three important parameters to be determined [53].

- Regularization parameter *C*: *C* is parameter for determining the tradeoff cost between minimizing training error and minimizing model complexity.
- Kernel parameter (*γ*): *γ* represents the parameter of the RBF kernel function.
- The tube size of e-insensitive loss function (*ε*): *ε* is the approximation accuracy placed on the training data points.

These parameters must be set correctly, in order to produce accurate estimation model. In this study, these parameters are determined through particle swarm optimization (PSO). The explanation on how PSO is used to estimate the parameters of SVR is given in Section 3.3.1.

### 3.2. Autoregressive Integrated Moving Average (ARIMA)

Autoregressive integrated moving average (ARIMA) model was introduced by Box and Jenkins and has become one of the most popular models in forecasting [17]. The ARIMA model is a stochastic model for time series forecasting where the future value of a variable is a linear function of past observations and random errors, expressed as

(8) yt=θ0+ϕ1yt−1+ϕ2yt−2+⋯+ϕpyt−p+εt −θ1εt−1−θ2εt−2−⋯−θqεt−q,

where *y*_*t*_ is the actual value and *ε*_*t*_ is the random error at time *t*, and *ϕ*_*i*_  (*i* = 1, 2,…, *p*) and *θ*_*j*_ (*j* = 0, 1, 2,…, *q*) are model parameters. Integers, *p* and *q* are referred to as order of the model and random errors, *ε*_*t*_, are assumed to be independently and identically distributed with a mean of zero and a constant variance of *σ* ^2^ [2]. 

ARIMA model is developed using Box-Jenkins methodology that involves the following three iterative steps [2].

*(i)  Model Identification.* At this step, data transformation should be done if necessary, to produce a stationary time series. Stationary time series is needed because the ARIMA model is developed with the assumption that the time series is stationary. Mean and autocorrelation structure are constant over time for stationary time series. Therefore, for a time series that exhibit trends and heteroscedasticity, differentiation and power transformation are necessary to change the time series to be stationary. Next, autocorrelation (ACF) and partial autocorrelation (PACF) are calculated from the data and compared to theoretical autocorrelation and partial autocorrelation for the various ARIMA models to identify the appropriate model form. The selected model is considered as a tentative model. Steps (ii) and (iii) in turn will determine whether the model is adequate [54]. 

*(ii) Parameter Estimation.* Once the tentative model is identified, parameters in ARIMA model can be estimated using the nonlinear least square procedure. 

*(iii) Diagnostic Checking*. The last step in model development is to check whether the model is adequate. Model assumptions about the errors must be met. Several diagnostic statistics and plots of the residual can be done to check the goodness of fit of the tentative model to the historical data. Among plots that can be very useful are histogram, normal probability plot, and time sequence plot. Residual autocorrelations should be small where chi-square test can be used to test the overall model adequacy. However, if the model is considered inadequate, a new tentative model should be identified and steps (ii) and (iii) will be repeated again.

Once a satisfactory model is produced, the three-step development process is no longer repeated and selected model will be used for forecasting purposes. In this study particle swarm optimization (PSO) as suggested by Asadi et al. [36] is used to estimate the parameters of ARIMA model. The explanation on how PSO is used to estimate the parameters of ARIMA model is given in Section 3.3.2.

### 3.3. Particle Swarm Optimization (PSO)

Particle swarm optimization (PSO) is one of stochastic optimization methods introduced by Kennedy and Eberhart [55]. This method is based on the natural evolution process which uses swarming strategies in bird flocking and fish schooling. PSO is a population-based which consists of particles. Initially, the particles are randomly generated. Each particle has a position and velocity, which represents a potential solution to a problem in *D*-dimensional space. The position and velocity of *i*th particle are denoted by *X*_*i*_ = (*x*_*i*1_, *x*_*i*2_,…, *x*_*iD*_) and *V*_*i*_ = (*v*_*i*1_, *v*_*i*2_,…, *v*_*iD*_), respectively. While solving the search problem, each particle explores the search space by moving in previous direction, its previous best particle (*pbest*), and the best solution for the entire population (*gbest*). The velocity and position of each particle are updated by using the following [56]:

(9) vij(t+1)=w·vij(t)+c1·rand1ij·(pbestij(t)−xij(t)) +c2·rand2ij·(gbestj(t)−xij(t)),

(10) $$\begin{array}{c} x_{ij}\left(t+1\right)=x_{ij}\left(t\right)+v_{ij}\left(t+1\right), \end{array}$$

where *v*_*ij*_(*t*) is the velocity of *i*th particle at iteration *t*, *x*_*ij*_(*t*) is the position of *i*th particle at iteration *t*, *j* = 1, 2,…, *D*, is the dimension of the search space, *w* is the inertia weight to balance the global and local search abilities of particles, rand1_*ij*_ and rand2_*ij*_ are two uniform random numbers generated independently within the range of [0, 1], *c*_1_ and *c*_2_ are two learning factors which control the influence of the social and cognitive components, *pbest*_*ij*_(*t*) is the best previous position yielding the best fitness value for *i*th particle at iteration *t*, and *gbest*_*j*_ is the global best particle by all particles at iteration *t*. After changing the position of the particle, the particle's fitness value is evaluated. The *pbest* and *gbest* are updated based on the current position of the particles. As this process is repeated, the whole population evolves towards the optimum solution.

The following are the steps in PSO implementation [57].

**Step 1**

Initialize the positions and velocities of all the particles randomly in the *D*-dimensional search space by uniform probability distribution function.

**Step 2**

Evaluate the fitness values of the particles.

**Step 3**

Update *pbest* for each particle; if the current fitness value of the particle is better than its *pbest* value, set the *pbest* equal to the current position of the particle.

**Step 4**

Update *gbest*; if the current fitness value of the particle is better than the *gbest* value, then set *gbest* equal to the current position of the particle.

**Step 5**

Update the velocity and position of each particle using (9) and (10), respectively.

**Step 6**

Repeat Steps 2 to 5, until stopping criteria are met, such as a sufficient good fitness value or a maximum number of iterations.

The explanations on how PSO is used to estimate the parameters of SVR and ARIMA models are given in Sections 3.3.1 and 3.3.2, respectively.

#### 3.3.1. PSO for SVR Parameters Estimation (PSOSVR)

Since there are three parameters to be estimated, the *i*th particle is represented by the three-dimensional vectors, *X*_*i*_ = (*x*_*i*1_, *x*_*i*2_, *x*_*i*3_) and *V*_*i*_ = (*v*_*i*1_, *v*_*i*2_, *v*_*i*3_), where the first, second, and third dimensions of the vectors refer to *C*, *γ*, and *ε*, respectively. In this study, the fitness is defined by *k*-fold cross-validation, where *k* = 5. In *k*-fold cross-validation, the training data set is divided into *k* subsets of equal size. One subset is used for validation. The regression function is built with a given set of parameters (*C*, *γ*, *ε*) using the remaining *k* − 1 subsets. The performance of the parameter set is measured by the root mean square error (RMSE) on the validation set. Each subset is used once for validation and the process is repeated *k* times. The average of RMSE on the validation set from 5 trials is used as a measure of fitness. The RMSE is defined as

(11) $$\begin{array}{c} \text{RMSE}=\sqrt{\frac{1}{n}\sum_{t=1}^{n}{\left(y_{t}-{\hat{y}}_{t}\right)}^{2},} \end{array}$$

where *n* is the number of validation data; *y*_*t*_ is the actual value and ${\hat{y}}_{t}$ is the predicted value. 

#### 3.3.2. PSO for ARIMA Parameters Estimation (PSOARIMA)

A hybrid model of PSO and ARIMA was proposed by Asadi et al. [36] to estimate the parameters of ARIMA model. This method is efficient for cases where inadequate historical data is available. The implementation of this method involves two main steps. First, an ARIMA model is generated by applying the Box and Jenkins method. Second, the PSO model is used to estimate the ARIMA parameters. The data set is divided into training and testing data set. The training data set is used to estimate the ARIMA model. However, testing data set is used to evaluate the estimation results. In this study, the fitness is defined by sum square error (SSE) as follows:

(12) $$\begin{array}{c} \text{SSE}=\sum_{t=1}^{n}{\left(y_{t}-{\hat{y}}_{t}\right)}^{2}, \end{array}$$

where *n* is the number of training data; *y*_*t*_ is the actual value and ${\hat{y}}_{t}$ is the predicted value. 

### 3.4. The Proposed Model

The proposed hybrid model consists of a nonlinear model, SVR, and a linear model, ARIMA. According to Zhang [2], it is reasonable to consider a time series as the composition of a linear autocorrelation structure and a nonlinear component, as

(13) $$\begin{array}{c} y_{t}=N_{t}+L_{t}, \end{array}$$

where *N*_*t*_ denotes the nonlinear component and *L*_*t*_ denotes the linear component. These two components are estimated from data using the following two steps. First, SVR model is used to model nonlinear components in the data. Second, the residual from the nonlinear model is modeled using linear model, ARIMA. Let *r*_*t*_ denote the residual which is represented by

(14) $$\begin{array}{c} r_{t}=y_{t}-{\hat{N}}_{t}. \end{array}$$

The residual represents linear components that cannot be modeled by SVR model. The SVR and ARIMA parameters are estimated by applying PSO, as described previously. Forecasting results from SVR and ARIMA models will be combined to represent the forecasting results of the proposed hybrid model. The combined forecast is shown by the formula (15)

(15) $$\begin{array}{c} {\hat{y}}_{t}={\hat{N}}_{t}+{\hat{L}}_{t}. \end{array}$$

**Figure 1:**
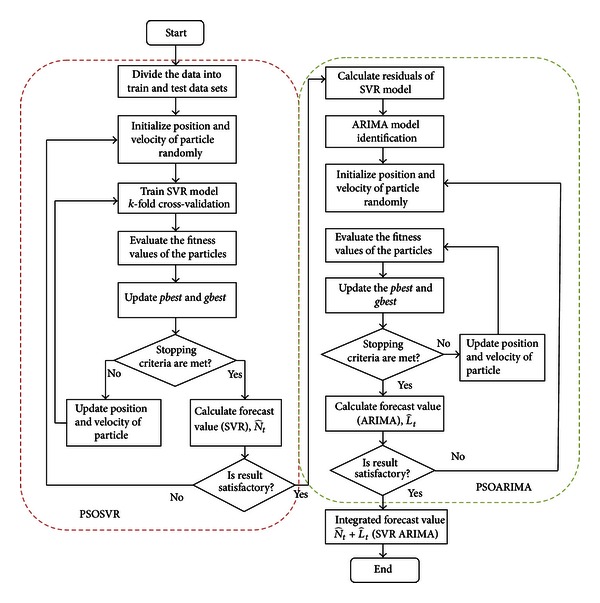
The Flow chart for the proposed hybrid PSOSVR and PSOARIMA model.

Figure 1 shows the flowchart for the proposed hybrid model, PSOSVR_PSOARIMA. 

## 4. Data Set and Model Evaluation

This section describes the data set used and the model evaluation carried out in this study.

### 4.1. Data Set

This study uses annual data of property crime rates, consumer price index for all urban consumers (Apparel), gross domestic product in United States natural log of billions of chained 2005 US Dollars, and unemployment rate (20 to 24 years) from 1960 to 2009 in United States. The crime rates are obtained from the Uniform Crime Reporting Statistics website (http://www.ucrdatatool.gov/), while economic indicators data are available on Economic Research Federal Reserve Bank of St. Louis website (http://research.stlouisfed.org/). Property crime data comprised property crime rate, vehicle rate, larceny-theft rate, and burglary rate. In addition to economic indicators, two crime indicators are also used in the model, which are one-year lagged property crime rate (PCR) and one-year lagged robbery rate. Data is divided into training and test data sets. The training data set is used to develop the models while the test data set is used to evaluate the forecasting performance of the models. In this study, 90 percent of the data will be used as training (1961 to 2004) and 10 percent is as test data set (2005 to 2009). 

### 4.2. Model Evaluation

The performance of the proposed hybrid model is evaluated using the test data set. The forecasting performance of the proposed hybrid model is evaluated using four types of evaluations.

*(i) Descriptive Statistics*. Graph of actual values and forecasting of testing data set is plotted in order to see the pattern of model predictions compared with the actual data patterns. A box plot diagram is used to check the error values. Box plot is used to see the dispersion of error values such as the position of median whether it is close to zero, and to ensure that there are no extreme values in error. 

*(ii) Quantitative Error Measurements*. Four types of quantitative error measurements are conducted, namely, root mean square error (RMSE), mean square error (MSE), mean absolute percentage error (MAPE), and mean absolute deviation (MAD). Formulas (16), (17), (18), and (19) are the equation for RMSE, MSE, MAPE, and MAD, respectively. Consider

(16) $$\begin{array}{c} \text{RMSE}=\sqrt{\frac{1}{n}\sum_{t=1}^{n}{\left(y_{t}-{\hat{y}}_{t}\right)}^{2}}, \end{array}$$

(17) $$\begin{array}{c} \text{MSE}=\frac{1}{n}\sum_{t=1}^{n}{\left(y_{t}-{\hat{y}}_{t}\right)}^{2}, \end{array}$$

(18) $$\begin{array}{c} \text{MAPE}=\sum_{t=1}^{n}\left|\frac{y_{t}-{\hat{y}}_{t}}{y_{t}}\right|\times \frac{100}{n}, \end{array}$$

(19) $$\begin{array}{c} \text{MAD}=\sum_{t=1}^{n}\frac{\left|y_{t}-{\hat{y}}_{t}\right|}{n}, \end{array}$$

where *n* is the number of test data; *y*_*t*_ is the actual value and ${\hat{y}}_{t}$ is the predicted value.

*(iii) Model Comparative Performance*. The performance of the proposed model is compared with individual model, PSOSVR, ARIMA, and PSOARIMA. A comparison with the hybrid model PSOSVR_ARIMA is also done to see the effects of PSO on the ARIMA model in the hybridization of SVR and ARIMA model. 

*(iv) Hypothesis Test*. The hypothesis test is performed to prove that there is no significant means difference between the forecasting values and the actual data. Paired sample *t*-test is used in this study. There are two hypotheses, the null hypothesis (*H*_0_) and the alternative hypothesis (*H*_1_). Let *μ*_1_ be the mean of actual data, *μ*_2_ the mean of forecasting values of the forecasting model, and (*μ*_1_ − *μ*_2_ = *μ*_*D*_) the difference of means. The hypothesis is that

(20) $$\begin{array}{c} H_{0}:\mu_{D}=0,\\ H_{1}:\mu_{D}\neq 0. \end{array}$$

The test statistic is shown by formula (21), which is *t* distributed with *n*_*D*_ − 1 degrees of freedom. Consider

(21) $$\begin{array}{c} t=\frac{{\overset{-}{y}}_{D}-\mu_{D}}{s_{D}/\sqrt{n_{D}}}, \end{array}$$

where ${\overset{-}{y}}_{D}$ is a sample mean difference, *s*_*D*_ is a sample standard deviation of the difference, and *n*_*D*_ is a sample size. The mean of forecasting values is equal to the mean of actual values, if the hypothesis test fails to reject the null hypothesis. It indicates that the model is appropriately used as forecasting model since it represents the real situation.

## 5. Determination Parameters of Model

In PSO algorithm, population size is set to 5, maximum number of iterations is set to 50 and the value of *c*_1_, *c*_2_ is set to 1. The inertia weight, *w*, initially is set to 1.4, and its value is decreased along with the iterations according to [58]

(22) $$\begin{array}{c} \text{Weight}=\frac{\left(\text{Weight}-0.4\right)*\left(\text{Maxiter}-\text{iter}\right)}{\text{Maxiter}}+0.4, \end{array}$$

where Maxiter is the maximum iteration and iter is the current iteration. The range values of SVR parameters (*C*, *γ*, *ε*) used in this study are shown in Table 1. Meanwhile, the searching range for ARIMA parameters is between −100 and 100.

The optimum parameter for SVR model obtained by PSO is (*C*, *γ*, *ε*) = (60.1924, 0.0625, 0.01). SVR model was developed using the optimum parameter which is then used to forecast the property crime rates using the training data and test data. After that, the residual which represents the difference between the actual value and prediction value was calculated. The residual of the training data was used to build the ARIMA model. Based on ACF and PACF, the appropriate model for the residuals is ARIMA (2,0,0), represented as

(23) $$\begin{array}{c} {\hat{e}}_{t}=a_{0}+a_{1}e_{t-1}+a_{2}e_{t-2}, \end{array}$$

where ${\hat{e}}_{t}$ is the forecast for period *t*, *e*_*t*−1_ and *e*_*t*−2_ are the residuals at time lags *t* − 1 and *t* − 2, and *a*_0_, *a*_1_, *a*_2_ are the coefficients to be estimated. Since the data varies around zero, the coefficient *a*_0_ is not required. Therefore, the coefficients to be estimated are *a*_1_ and *a*_2_. PSO was applied to estimate the value of the coefficients. The *i*th particle is represented by the two-dimensional vectors, *X*_*i*_ = (*x*_*i*1_, *x*_*i*2_) and *V*_*i*_ = (*v*_*i*1_, *v*_*i*2_), where the first and second dimensions of the vectors refer to *a*_1_ and *a*_2_, respectively. After using the PSO, the coefficients were estimated and the ARIMA model is shown as

(24) $$\begin{array}{c} {\hat{e}}_{t}=0.3445e_{t-1}-0.1916e_{t-2}. \end{array}$$

Next, the ARIMA model obtained is used to forecast the residual from the SVR model. The forecast results from SVR and ARIMA models are combined, to represent forecast results of the proposed hybrid model. 

## 6. Analysis of the Results

**Figure 2:**
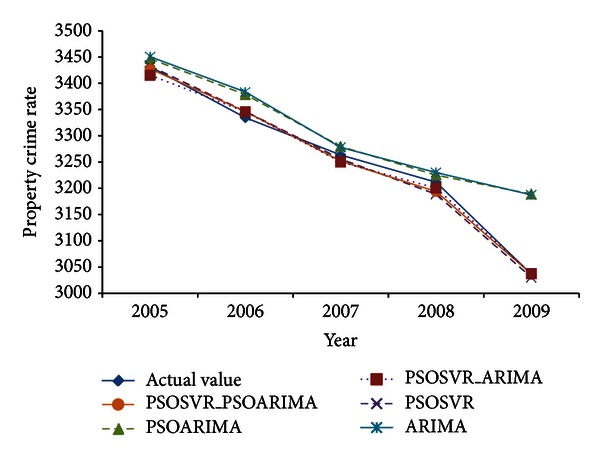
Forecasting of test data set.

**Figure 3:**
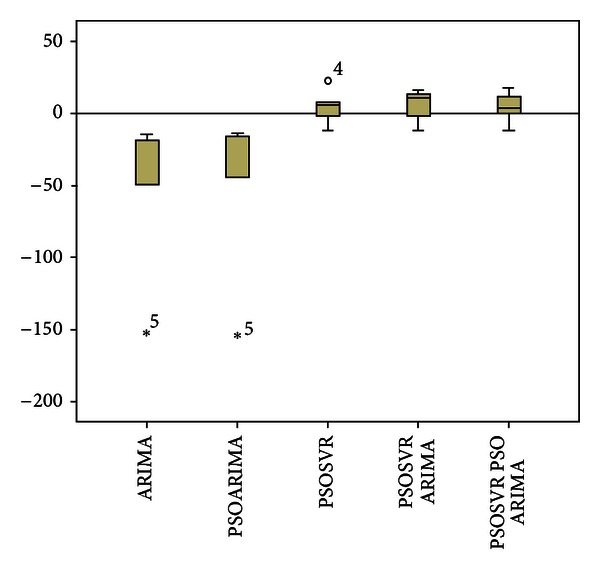
Forecasting Errors of test data set.

Table 2 and Figure 2 show the comparison of actual values and forecast values of property crime rates for test data set. PSOSVR_PSOARIMA, PSOSVR, and PSOSVR_ARIMA were found to predict closer to the actual value and have a similar pattern with the actual data. Meanwhile, ARIMA and PSOARIMA show unsatisfactory forecasting performance with the predicted values slightly higher than the actual value.  Figure 3 shows box plot of forecast errors for testing data set. Based on the box plot, there is an outlier in the error of the PSOSVR model, while for ARIMA and PSOARIMA models, respectively, there is an extreme error value. The hybrid models, PSOSVR_PSOARIMA and PSOSVR_ARIMA, show better forecasting performance than the individual models, with no outliers or extreme error values. The proposed hybrid model was found to have the least forecast errors with a median closer to zero as compared to other forecasting models. 

Table 3 shows the RMSE, MSE, MAPE, and MAD of the proposed hybrid model in comparison to the individual models, ARIMA, PSOARIMA, PSOSVR, and hybrid model, PSOSVR_ARIMA. The linear models, ARIMA and PSOARIMA, show poor performance with a relatively large error values in comparison to nonlinear model, PSOSVR and hybrid models. On the other hand, the nonlinear model, PSOSVR, shows a comparable performance to hybrid models with a slight difference in error values. The proposed hybrid model is found to have smaller errors than the individual models and PSOSVR_ARIMA. In addition, PSO is found to be able to improve the performance of the ARIMA model in the hybridization of SVR and ARIMA model. The results have shown that the use of PSO on ARIMA model is capable of improving the performance of the hybrid model PSOSVR_ARIMA. Therefore, PSOARIMA is suitable to be applied on small data set.

Table 4 shows the results of paired samples *t*-test. Paired samples *t*-test is used to compare the actual data with the five forecasting models that have been developed, namely, ARIMA, PSOARIMA, PSOSVR, PSOSVR_ARIMA, and PSOSVR_PSOARIMA. The test is to ensure that there is no statistically significant difference of means between the actual data and the forecast values from the forecasting models. The results show that between the actual data and the forecast values from the forecasting models, the *P* value >0.05 (0.124, 0.145, 0.463, 0.333, and 0.443). The difference between the upper and lower values for 95% interval is range between negatives and positives values. The results indicate that the hypothesis test fails to reject the null hypothesis. This implies that there is no statistically significant difference in the means between the actual data and the forecasting models. However, PSOSVR_PSOARIMA shows the smallest mean, standard deviation, and standard error mean as compared to other forecasting models. Therefore, the assumption can be made that PSOSVR_PSOARIMA is able to give better forecasting performance in comparison to other forecasting models.

Based on model evaluation that has been carried out, we can conclude that the individual models, ARIMA, PSOARIMA, and PSOSVR, are not sufficient to model the property crime rates. The hybrid models are more suitable to be employed as forecasting model for property crime rates, where the proposed hybrid model shows the best forecasting performance. These results indicate that there are linear and nonlinear components in property crime rates. Therefore, the use of linear and nonlinear models individually is not sufficient for modeling the property crime rates. However, the optimal parameters are very important for ensuring the accuracy of SVR models. The use of PSO has facilitated the searching process for the optimal parameters of SVR model, thus able to produce more accurate models. Results of this study also demonstrated that the use of PSO in estimating the parameters of ARIMA model was able to improve the accuracy of the ARIMA model for small data sets. 

## 7. Conclusions

This paper proposes a time series model for crime rates forecasting. The proposed model is a hybrid model that combines the nonlinear model, SVR, with linear model, ARIMA. The proposed model was used to predict the property crime rates. The PSO is used to estimate the parameters of SVR and ARIMA models. Economic indicators are used as inputs to the proposed model. The economic indicators used are gross domestic product, unemployment rate, and consumer price index. The experimental results have found that the proposed model, PSOSVR_PSOARIMA, can produce smaller errors as compared to the individual models and hybrid model, PSOSVR_ARIMA. In conclusion, it can be concluded that the proposed hybrid model is an acceptable model to be applied in the crime rates forecasting.

## Figures and Tables

**Table 1:** The range values for the parameters (*C*, *γ*, *ε*).

Parameters	Range values
*C *	2^−1^ to 2^7^
*γ*	2^−4^ to 2^2^
*ε*	0.01 to 0.05

**Table 2:** Comparison of actual value and forecast value of property crime rates.

Year	Actual value of property crime rates	Forecast value of property crime rates				
		Individual models			Hybrid models	
		ARIMA	PSOARIMA	PSOSVR	PSOSVR ARIMA	PSOSVR PSOARIMA
2005	3431.5	3450.674	3446.673	3432.788	3415.503	3427.515
2006	3334.5	3383.523	3378.434	3346.533	3345.65	3346.024
2007	3263.5	3277.728	3279.746	3255.626	3250.134	3251.728
2008	3211.5	3230.002	3225.111	3188.747	3200.334	3193.765
2009	3036.1	3187.335	3189.071	3030.249	3037.259	3036.579

**Table 3:** Comparison of errors.

Model	RMSE	MSE	MAPE	MAD
ARIMA	72.371	5237.568	1.604	50.433
PSOARIMA	72.125	5201.967	1.544	48.387
PSOSVR	12.332	152.077	0.308	9.960
PSOSVR_ARIMA	11.704	136.980	0.319	10.568
PSOSVR_PSOARIMA	10.973	120.408	0.278	9.099

**Table 4:** Paired samples test.

	Paired differences							
	Mean	Std. deviation	Std. error mean	95% confidence interval of the difference		*t *	df	Sig. (2-tailed)
				Lower	Upper			
Pair 1 PCR-ARIMA	−50.43259	58.03148	25.95247	−122.48819	21.62301	−1.943	4	0.124
Pair 2 PCR-PSOARIMA	−48.38690	59.74264	26.74264	−122.63637	25.86257	−1.809	4	0.145
Pair 3 PCR-PSOSVR	4.63141	5.71462	5.71462	−11.23491	20.49773	0.810	4	0.463
Pair 4 PCR-PSOSVR_ARIMA	5.64408	5.12651	5.12651	−8.58939	19.87756	1.101	4	0.333
Pair 5 PCR-PSOSVR_PSOARIMA	4.29777	5.04821	5.04821	−9.71831	18.31385	0.851	4	0.443

Legend: Pair 1: actual data and ARIMA; Pair 2: actual data and PSOARIMA; Pair 3: actual data and PSOSVR; Pair 4: actual data and PSOSVR_ARIMA; Pair 5: actual data and PSOSVR_PSOARIMA.
